# Physical and psychological health outcomes of a sitting light volleyball intervention program on adults with physical disabilities: a non-randomized controlled pre-post study

**DOI:** 10.1186/s13102-021-00328-7

**Published:** 2021-08-28

**Authors:** Ka-Man Leung, Pak-Kwong Chung, William Chu, Kwok Ng

**Affiliations:** 1 Department of Health and Physical Education, Education University of Hong Kong, Tai Po, Hong Kong, China; 2 Department of Sport, Physical Education and Health, Hong Kong Baptist University, Hong Kong, China; 3 School of Educational Sciences and Psychology, University of Eastern Finland, Kuopio, Finland; 4 Physical Activity for Health, Department of Physical Education and Sport Sciences, University of Limerick, Limerick, Ireland

**Keywords:** Adaptive physical activity, Sport, Special population

## Abstract

**Background:**

People with physical disabilities (PWPD) have limited opportunities to participate in sport activities. Sitting light volleyball (SLVB) is an adapted sport that combines light volleyball and paralympic sitting volleyball. This study examined the effectiveness of an SLVB intervention program to improve the physical and psychological health outcomes of PWPD in Hong Kong, China.

**Methods:**

Thirty-two PWPD [13 women; SLVB group, n = 18; control group (CG), n = 14] with an average age of 48.89 years (SD = 14.42 years) participated in a 16-week intervention consisting of basic SLVB skills, and they also received instructions on the required posture, team tactics, and SLVB rules. Physical (i.e., muscular strength, muscular endurance, body composition, flexibility, and aerobic endurance) and psychological (i.e., physical activity enjoyment and quality of life) health outcomes were measured before and after the intervention.

**Results:**

Individuals in the SLVB group exhibited statistically significant improvements in cardiovascular endurance [*F*(1,29) = 4.23, *p* = .049], body composition [*F*(1,23) = 6.67, *p* = .017], and physical activity enjoyment [*F*(1,29) = 16.94, *p* = .001] compared with adults in the CG.

**Conclusions:**

Participating in SLVB has physical and psychological benefits for adults with physical disabilities in this study.

*Registration number of trial registry*: The trial is registered at chictr.org.cn, number ChiCTR2000032971 on 17/05/2020.

## Introduction

### Background

In Hong Kong, among the 578,600 persons with disabilities, approximately 320,500 individuals (55%) have restrictions in body movement [1]. The proportion of people with restrictions in body movement increased from 2.72% in 2007 to 4.47% in 2013 [1]. This increase is expected to continue, partly due to the recent aging population trend in Hong Kong. Researchers have observed that people with disabilities (PWD) have poorer health, a higher risk of early mortality, and a higher incidence of both mental and physical illnesses [2, 3]. PWD also have lower physical fitness, poorer quality of life, and lower rates of participation in physical activities (PA) compared with their peers without disabilities [4]. These lower PA rates, fitness level, and quality of life are of great concern to society because they are associated with hypokinetic health problems (i.e., diseases resulting from the lack of exercise) such as functional limitations and social isolation [2, 3]. This health problem is compounded when PWD become deconditioned and sedentary as they age.

These health concerns are in fact linked to our service gap that exists in the provision of sports programs and interventions for people with physical disabilities [PWPD, 5–8]. In Hong Kong, a consultancy study [9] on sports programs for people with physical disabilities (PWPD) concluded that nongovernment organizations, such as Hong Kong Federation of Handicapped Youth (HKFHY) provide a lack of sport activities for members with motor disabilities. The staff of these NGOs reported their members have insufficient opportunities for participation in sport or PA. This result was particularly prevalent when compared with the opportunities provided to people with other types of disabilities, such as intellectual disability [9]. Similarly, van der Ploeg, and colleagues [10] found that planned and structured activities for PWPD are lacking, particularly after their rehabilitation programs, and Jaarsma and Smith [11] identified more trials based on community led research is needed.

### Light volleyball and older adults

Light volleyball (LVB), also known as “gas volleyball” in mainland China, is a popular recreational activity among older adults in China [12]. Studies in China [13] and Hong Kong [14] have indicated that older adults gain physical and psychological health benefits from regular LVB practice. With the positive results of our mentioned intervention, we hypothesised that LVB would also bring positive health impact (e.g., physical and psychological) to other populations with low levels of physical fitness, such as PWPD.

### Sitting LVB and PWPD

In this study, we extend PI’s previous work [14] aimed at promoting LVB programs for PWPD. Sitting light volleyball (SLVB) is a version of Paralympic sitting Volleyball (SVB), which combines SVB and LVB, may be a suitable PA for PWPD who have relatively low fitness levels and sport competence levels. SLVB is similar to SVB that SLVB is played in a sitting position. Participants are required to move in different directions by using their hands to move and slide across the playing court when receiving, blocking, and serving the ball. SLVB is inclusive and enables players with and without PDs to play together and interactively. It also entails the ball bouncing no more than once in each pass. When a team wins a rally, irrespective of whether the team has the right to serve, the teams rotate (clockwise) before the next service. Throughout every game, participants used their upper limbs to support and drag their bodies to complete the rotation requirement. Moreover, this noncontact team sport can be played on a standard badminton court, which is smaller than a volleyball court. Sport facilities in land-scarce Hong Kong tend to be limited, and the use of a smaller court increases the sites at which SLVB can be played by PWPD. The relatively lightweight and soft cover of the ball make playing the ball off the forearms easier; additionally, the SLVB with lower velocity [15] ball may be easier to control and more playable (more enjoyable), and SLVB is also easier to learn, making it accessible to those with motor disabilities and muscle degradation [14].

### Objectives

There is an increasing proportion of PWPD in Hong Kong [1], who have decreased levels of fitness, low participation in PA, lower level of quality of life, and decreased opportunities for participation in various sports [9]. Our study examined the physical (i.e., physical fitness) and psychological (i.e., physical activity enjoyment) health outcomes of a SLVB intervention program on PWPD in Hong Kong. We hypothesized that the SLVB group should significantly improve physical and psychological health measures of PWPD compared to control group.

## Methods

### Study design and participants

**Fig. 1:**
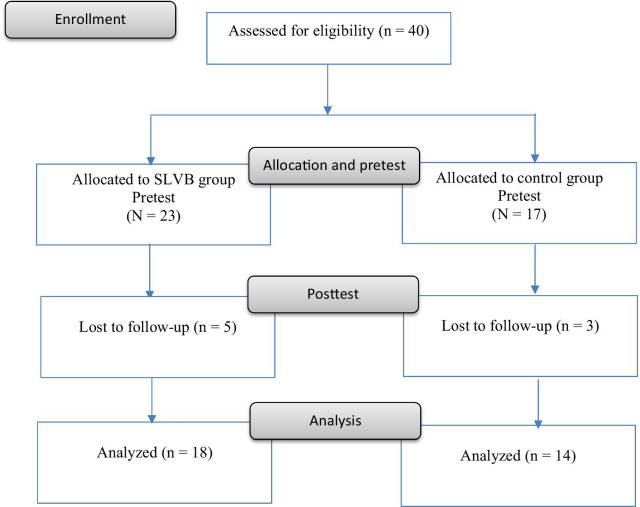
Flow chart of data collection before and after the intervention

We applied a non-randomized controlled pre-post study design. Participants were divided into two groups: an SLVB group and a control group (CG), initially in 1:1 ratio. However, due to difficulties recruiting participants in control group, the allocation ratio was not balanced in the groups. The study flowchart is illustrated in Fig. 1. Body composition, upper body muscular strength, muscular endurance, cardiovascular endurance, flexibility, PA enjoyment, and quality of life were measured as outcome variables. The inclusion criteria were as follows: (a) adults aged ≥ 18 years, (b) PWPD registered in the Central Registry of Rehabilitation, (c) presence of at least one functional arm, (d) no diagnosis of cognitive impairment, and (e) no participation in a structured PA program in the 6 months preceding the study. The exclusion criteria were as follows: (a) history of cardiovascular disease that would hinder study participation; (b) severe body pains, dizziness, and uncontrollable high blood pressure (> 160/100 mmHg); (c) seeing and hearing difficulties; (d) physician disapproval to participate in the study due to a limiting medical and physical conditions; and (e) inability to communicate in Chinese. All eligible participants received a form providing a brief description of the study and were asked to sign a consent form. Research staff responded to any questions from participants about the study. This study was approved by the Institutional Review Board of University (Human Research Ethics Committee of Education University of Hong Kong, A2019-2020–0303).

Based on the study of Van den Berg-Emons et al. [16] that the effect size (Cohen’s d) for daily PA as energy expenditure after an aerobic exercise program in young people with cerebral palsy was 1.05. Our power analysis was conducted using G power [version 3.1.9.2 [17]] and indicated a total sample (Cohen’s d) of 28 (14 per group) was required to ensure a power of 80% and an alpha of .05. Therefore, we recruited at least 18 participants per group (SLVB group and CG) in expectation of a dropout rate of 20% [18].

#### Intervention program

Participants underwent our intervention program for 16 weeks in sport complex of Hong Kong Baptist University between May and September 2018. The content of the SLVB intervention was based on an LVB program designed specifically for older adults [14]. In short, the content included basic skills and posture of SLVB, its team tactics, and its rules and regulations. This SLVB intervention program comprised 32 training sessions, with two 90-min sessions conducted every week. Our program was conducted by registered coaches from the Hong Kong Light Volleyball Association. Each session began with a 10-min warm-up, drills, game session, and concluded with a 5-min stretch and cool-down. During the intervention period, CG participants were instructed to continue with their regular daily routines.

#### Safety precautions

Based on professional advice from our partnered physiotherapist, the movements in the SLVB intervention were matched with our participants’ physical conditions. The physiotherapist provided feedback on the safety protocol, content, and teaching effectiveness of our SLVB intervention. Participants were given two elbow pads for protection against possible contusions (if needed). Pulse oximeters were used to monitor participants’ exercise intensity during the intervention. Notably, throughout our study, appropriate warm-ups and cool-downs were conducted, and participants were given one 10-min rest in the middle of a training session, as suggested previously [19].

#### Procedures

Participants were recruited by an insider of HKFHY/external partner who had access to individual members of an academy for PWPD during March – May 2018. This insider posted messages on a WhatsApp group and placed advertisements in their bimonthly magazine. PWPD who expressed interest in participating in the study were informed about the confidentiality of personal data and voluntary participation in the study. In addition, trained test administrators, who conducted the tests, were blinded to group assignment.

At the first testing session prior to the pretest, participants were briefed on the aims, procedures, and expectations of the intervention program. They underwent screening tests (including checks on their history of cardiovascular disease). After informed consent was obtained from participants, data collection was conducted by trained test administrators. The administrators described the tests to participants and explained what the tests aimed to assess [20]. Participants were administered the fitness tests in an order suggested by a previous study [21] to maximize recovery and minimize fatigue. The test items were administered to one participant at a time. Moreover, participants completed a questionnaire, and their heights and weights were measured [20].

After testing, participants were allocated to the SLVB group or CG. The intervention began the following week. After 16 weeks (at the end of the intervention), participants completed the posttest using the same protocol as used in the pretest. The pretest and posttest were conducted in accordance with the recommendations for administering the Brockport Physical Fitness Test (BPFT) [20]. Only participants with at least 80% attendance in the SLVB intervention were allowed to complete posttest measures. All participants received a supermarket cash voucher of HK$100 as an incentive for their participation.

#### Measures

We used the Brockport physical fitness test, BPFT [20] to measure the physical fitness attributes of participants. The components of the physical fitness tests adopted for this study included the dominant grip strength test (to measure upper body muscular strength), dumbbell press test (to measure upper body muscular endurance), skinfold test (at the scapula; to measure body composition, such as fat ratio), and shoulder stretch test (to measure flexibility). The BPFT has been demonstrated to be a valid and reliable physical fitness test for people with different disabilities [22].

We also used the multistage field test (MFT) [23] to measure participants’ aerobic endurance. The MFT was performed according to auditory instructions: participants were directed to move around in a wheelchair around a 15-m^2^ octagonal course delimited by cones. The velocity with which the participants moved in a wheelchair was increased every minute until they were exhausted. The total distance travelled was recorded. The validity (predictive to maximum oxygen uptake: *r* = .59) and reliability (test–retest reliability: ICC = .99) of the MFT have been demonstrated in a study involving adults using wheelchairs [23]. In line with an assessment by Vanderthommen et al. [23], our participants could use their personal or sports wheelchair for both the pretest and posttest assessments.

In addition to administering the fitness tests, we measured participants’ PA enjoyment. The short, Chinese version of the Physical Activity Enjoyment Scale (PACES) [24] was used to measure PA enjoyment in this study. The eight-item PACES has been demonstrated to be a reliable (test–retest reliability: ICC = .614) and valid (convergent validity: *r* = .43) instrument for assessing PA enjoyment among Chinese adults [24]. Seven polarised rating items include “I find it pleasurable” (one end) to “I find it unpleasurable” (the other end). The mean score of the eight items was calculated to determine the PA enjoyment score.

#### Data analysis

Data were analyzed using SPSS 26.0. The *t* test was used to evaluate baseline differences between the groups. A series of 2 (group: intervention group vs control group) by 2 (time: pretest vs posttest) ANCOVA for each outcome physical and psychological attribute were used to examine the effects of the intervention on measures of physical and psychological attributes in the groups. Participants’ body mass index that correlated with these attributes were used as covariate. All effect sizes are reported as partial η^2^ and they are interpreted as medium: η^2^ = .06 and large: η^2^ = .14 [25]. The alpha was set at *p* < .05 for all statistical tests.

## Results

### Demographic characteristics

The sociodemographic characteristics of participants are presented in Table 1. Forty participants (SLVB group, n = 23; men, n = 7; CG, n = 17; men, n = 10) completed the pretest and our SLVB intervention. Eight individuals (five from the SLVB group and three from the CG) did not participate in posttest measurements or they withdrew from the study before completion, leaving 32 participants (SLVB group, n = 18; CG, n = 14) with analyzable data. Excluding data from individuals who withdrew from the SLVB group, the average attendance rate was 71.81%. The mean age was 48.89 years [standard deviation (SD) = 14.42]. Over 50% of them aged between 50–58 years old. Approximately 40% of participants were women. Most participants had secondary (68.75%) or tertiary (25%) school education and a family income of HK$30,000–HK$69,999 (68.75%). Approximately half were either homemakers (18.75%) or retirees (37.5%). The most common health conditions were poliomyelitis (n = 28), limb deficiency (n = 4), cerebral palsy (n = 2), and others (n = 6).

**Table 1:** Socio-demographic characteristics of participants

	SLVB	Control
Age (mean ± SD)	52.28 (8.86)	45.64 (17.39)
Gender		
Female	7	6
Male	11	8
Occupation		
Full time job	7	3
Searching for job	0	1
Housewife	5	1
Retired	5	7
Part-time job	1	2
Education level		
Primary education	1	1
Secondary education	12	10
Tertiary education	5	3
Family income (in HKD)		
90,000 or above	2	2
70,000–89,999	1	3
50,000–69,999	7	6
30,000–49,999	7	2
10,000–29,999	0	1
5000–9999	1	0
4999 or below		
Body Mass Index, kg/m^2^	23.60 (3.97)	23.28 (5.74)

SLVB, Sitting Light Volleyball group; CG, Control group

### Outcome measures

The differences in pretest scores between the CG and SLVB group were not statistically different for all outcome measures, except for PA enjoyment (*t* (30) = 2.07, *p* = .047). Slightly higher levels of enjoyment were reported by the SLVB group (5.21, SD = .96) than the CG (4.58, SD = .70).

The means and SDs of physical and psychological measures of the groups at pretests and posttest are presented in Table 2. After the intervention, the SLVB group had statistically significant lower adjusted fat mass levels [Triceps, *F*(1,29) = 4.17, *p* = .050, η^2^ = .126] and higher adjusted aerobic endurance levels [*F*(1,29) = 4.27, *p* = .048, η^2^ = .128] compared with the CG. There were better improvements in fat mass level and aerobic endurance level over times in SLVB intervention group, comparing to control group. No significant interactive effects were found in other physical measures (See Table 2).

**Table 2:** Means and standard deviations for measures in groups at pretests and posttests

Measures	CG (N = 14)		SLVB (N = 18)		*F*	*p*
	Pretest	Posttest	Pretest	Posttest		
Skinfold test (mm)	15.94 (1.35)	14.38 (.77)	19.27 (1.19)	14.23 (.68)	4.17	.050*
Handgrip Strength test						
Dominant hand	25.94 (2.43)	25.43 (2.58)	27.47 (2.14)	28.16 (2.28)	1.15	.293
Non-dominant hand	23.46 (2.72)	24.22 (3.00)	26.14 (2.39)	28.47 (2.64)	1.77	.194
Dumbbell Press (frequency)						
Dominant hand	18.40 (3.39)	18.57 (3.52)	23.80 (2.99)	28.06 (3.11)	3.93	.057
Non-dominant hand	13.58 (3.54)	14.97 (3.76)	21.05 (3.12)	23.24 (3.31)	.12	.728
Multistage Field Test (m)	340.69 (71.79)	352.18 (86.13)	317.42(63.31)	458.97(75.95)	4.27	.048*
Shoulder stretch (mm)						
Right	− 127.10 (34.52)	− 131.98 (37.66)	− 113.37 (30.44)	− 96.79 (33.21)	.49	.489
Left	− 162.49 (30.84)	− 174.62 (39.15)	− 164.17 (27.19)	− 110.29 (34.53)	1.52	.227
PACES (Score)	4.57 (.22)	4.65 (.23)	5.22 (.20)	6.10 (.20)	5.34	.028*

*p < .05; **p < .01; SLVB, Sitting Light Volleyball group; CG, Control Group. Adjusted via ANCOVA for BMI

Psychologically, a significant interaction effect was found for PA enjoyment [F(1,28) = 5.34, *p* = .028, partial η^2^ = .156]. Intervention group showed greater improvement in PA enjoyment over time compared with the control group.

## Discussion

This study aimed to examine the effectiveness of an SLVB intervention program for the physical and psychological health outcomes of PWPD in Hong Kong. Following a 16-week intervention that involved participation in SLVB, compared with CG group, there were improvements in cardiovascular endurance, body composition (lower fat mass), and PA enjoyment in SLVB group. There were no differences in flexibility, upper body strength, and endurance between groups. Therefore, our main hypothesis was partially supported; the SLVB intervention was associated with significant improvements in physical (e.g., cardiovascular endurance and body composition) and psychological health (e.g., PA enjoyment) attributes in PWPD, compared to PWPD not exposed to the intervention.

### Improvements in physical health

Similar to participants in traditional indoor volleyball [26] or SVB [27], participants in SLVB exhibited significant improvements in cardiovascular endurance and body composition at the end of the intervention. Improvements in cardiovascular endurance were expected due to the movements involved in playing volleyball in the sitting position, such as sliding on one’s buttocks and using (particularly upper) limbs [28].

The duration of the sessions in the SLVB programme exceeded the World Health Organization PA recommendations [29], in that adults should participate in at least 150 min of moderate-intensity aerobic activity weekly. This may be another explanation for the improvements in cardiovascular endurance observed among participants in the SLVB group. Increased cardiovascular endurance resulting from the intervention may have contributed to lower fat mass of the SLVB group. This result partially corroborates prior findings that exercises that improve cardiovascular endurance, improve fat distribution, and reduce overweight and obesity [30].

*Lack of improvements in flexibility, upper body muscular strength, and muscular endurance.* A previous study [27] confirmed the relationships between flexibility, muscular strength, muscular endurance, and performance of SVB skills. For example, serving and blocking were correlated with flexibility (i.e., shoulder stretch test), whereas defense, service, and grip strength were correlated. In addition, higher levels of muscular endurance predicted higher performance in serving, blocking, and overall performance of SVB players. Another study [31] also demonstrated that vertical reach in the seated position is one of the most crucial factors in the performance of SVB athletes. However, our findings contrast with these results. In the present study, inadequate blocking and spiking during the intervention likely contributed to the nonsignificant improvement in the flexibility of participants. Even though we had a training session for practicing both spiking and serving (underarm or overhead serving), most participants did not perform much spiking, and they often served using the underhand technique, which requires lower upper body muscular strength more than the overhead movement, during game sessions in our intervention. In addition, during game sessions in the intervention, underarm service was dominant among participants with a lower degree of skill. In addition, tests such as the grip strength test (forearm strength), might not fully reflect participant’s upper body muscle endurance improvement resulting from the intervention. Other SLVB movement-specific measurements should be considered in the future. Altogether, these factors explain the nonsignificant improvement in upper body muscular ability and flexibility.

### Improvement in PA enjoyment

In this study, compared with the CG, the SLVB group reported greater PA enjoyment. These results are consistent with our previous results [14] and those of another study applying water volleyball, another adapted version of volleyball, to older adults [32]. SLVB is a team sport in which players cooperate and work with their teammates. Players must collaborate and communicate with their team members in the processes of passing, setting, and spiking. Volleyball is generally played with high team spirits and may create a greater sense of enjoyment [33], along with improved competence, physical self-esteem, and the development of positive habits as was reported in previous studies [34–36]. In addition to the dynamics between participants themselves or the feelings of participants toward the activity, the coaches’ encouragement and feedback provided in the intervention might also contribute to participants’ relatedness, as suggested by self-determination theory [37], and consequently enhance the likelihood of PA enjoyment. All the above might explain the greater PA enjoyment of the SLVB group.

In sum, our above significant results may be because we used standardized fitness measurements, center- and group-based interventions, and professional guidance from intervention coaches. Notably, the adherence rate for the intervention program was high (71.81%). Approximately 80% of participants completed both pretest and posttest measures. Thus, our observed 20% dropout rate corresponded to our expected dropout rate. This expected dropout rate had been suggested by Hicks et al. [18], who studied long-term exercise training in people with spinal cord injury. This low dropout rate might be because our intervention entailed fun, self-efficacy, and social contacts, which encouraged participation, and this also helped overcome barriers such as the lack of opportunities for participation in sports and accessibility problems, as suggested in a previous study [38].

### Limitations and recommendations for further research

Our study has some limitations. First, this was a study examining the effects of the SLVB intervention on the health of a small group of PWPD from one nongovernmental organisation. Also, given evidence that SLVB was beneficial to the physical and psychological health of general PWPD, evidence is warranted to test its impact in a larger group of PWPD and with specific type of PWPD. All these will enhance the generalizability of the intervention findings. Second, SLVB movement-specific measurements should be considered in future interventions; for example, measuring aerobic endurance while moving on the floor using hands. Third, because of resource constraints, no follow-up was conducted. Fourth, about 8 of 40 participants did not complete our posttest measures. Therefore, future studies should include follow-up assessment with a more effective plan to monitor adherence to the intervention and changes in physical and psychological measures through study designs such as the wait-list control trial design. Fifth, in addition to applying quantitative methods to examine the effects of SLVB on health among PWPD, researchers may need to adopt a mixed-methods approach to comprehensively examine both the training effects and participants’ experience in a future SLVB interventions program.

## Conclusion and practical implication

This is the first study to examine the effects of an intervention involving an adapted sport, SLVB, on the health outcomes of PWPD in Hong Kong. Our results showed that SLVB was beneficial to physical health (i.e., cardiovascular endurance and body composition) and psychological heath (i.e., PA enjoyment) among PWPD. Randomized control trials should be conducted to further investigate the effects of SLVB on health outcomes in the community and across multiple service centers. SLVB is a safe noncontact team sport with an easy-to-control ball. Moreover, it is playable on badminton courts, which avoids the problem of space constraints. Therefore, SLVB is accessible to people with motor disabilities and muscle degradation in the community. Our study therefore provides evidence-based information to the government and related practitioners for the promotion of adapted PA (such as SLVB) among PWPD.

## Data Availability

The datasets generated and analyzed during the current study are not publicly available due to ethical restrictions but are available from the corresponding author upon reasonable request.

## Abbreviations

PWPD: People with physical disabilities (PWPD)
SLVB: Sitting light volleyball
CG: Control group
PA: Physical activities
LVB: Light volleyball
SVB: Sitting volleyball
BPFT: Brockport Physical Fitness Test
MFT: Multistage field test
PACES: Physical Activity Enjoyment Scale (PACES)
SF-36: 36-Item Short Form Health Survey
ANCOVA: Analysis of covariance
